# Editorial: Microorganisms in agricultural soil: advances and challenges of biological health

**DOI:** 10.3389/fmicb.2025.1752541

**Published:** 2025-12-15

**Authors:** Yanlong Chen, Xiaomeng Wei, Cui Li, Cong Wang

**Affiliations:** 1 School of Ecology and Environment, Northwestern Polytechnical University, Xi'an, Shaanxi, China; 2 College of Natural Resources and Environment, Northwest A&F University, Xianyang, China; 3 Xi'an Botanical Garden of Shaanxi Province, Institute of Botany of Shaanxi Province, Xi'an, China; 4 Guangxi Key Laboratory of Forest Ecology and Conservation, State Key Laboratory for Conservation and Utilization of Subtropical Agro-Bioresources, College of Forestry, Guangxi University, Nanning, China; 5 Department of Land, Air and Water Resources, University of California, Davis, Davis, CA, United States

**Keywords:** soil degradation, microbial community structure, microbial functions, network, community assembly

The concept of soil biological health has evolved from a specialized interest to a cornerstone of sustainable agriculture. A healthy agricultural soil is not merely an inert substrate but a dynamic, living ecosystem. The intricate activities of its microbial inhabitants drive essential processes, including nutrient cycling, carbon sequestration, pollutant degradation, and plant pathogen suppression. Yet, this biological foundation is increasingly threatened by anthropogenic activities, leading to degradation marked by reduced fertility, elevated greenhouse gas emissions, and accumulation of contaminants. This Research Topic, “*Microorganisms in agricultural soil: advances and challenges of biological health*”, was initiated to generate new insights into these pressing issues and explore innovative pathways for soil restoration and sustainable management. This Research Topic collected 22 submissions, of which 13 were accepted after peer review. The published articles provide a multi-faceted exploration of soil microbiome dynamics.

The study by Wang X. et al. offers a paradigm-shifting perspective by underscoring the often-overlooked role of microelements. Their research in loquat orchards reveals that elements such as available magnesium and copper exert a stronger influence on the structure of bacterial, fungal, and protistan communities than geographical distance or basic soil properties including macronutrients. Crucially, this work links microelement-driven microbial shifts directly to fruit quality, highlighting the importance of precision micronutrient management for both soil and crop health.

Building on the theme of elemental cycling, Jiang et al. examine the critical role of soil microorganisms in selenium biogeochemistry. Their synthesis details microbial processes, including reduction, oxidation, methylation, and volatilization, that govern selenium speciation. These transformations are vital not only for detoxifying selenium-contaminated environments but also for enhancing the bioavailability of this essential micronutrient in plants, thereby linking soil microbial activity to human nutrition through the food chain.

Shifting from elemental cycles to agricultural practice, Duan et al. decipher the microbial and metabolic mechanisms underlying continuous cropping obstacles in the medicinal herb *Gastrodia elata*. Using integrated metabolomic and metabarcoding approaches, they identify key bacterial genera (e.g., *Massilia, Burkholderia, Caballeronia, Paraburkholderia*, and *Dyella*) and specific soil metabolites such as 4-hydroxy-benzenemethanol as indicators of soil sickness. Their study provides a mechanistic understanding of how monoculture disrupts soil ecosystems, offering insights for targeted management strategies.

Liu et al. demonstrate how tobacco *Fusarium* wilt disease (FWD) reshapes the rhizosphere microbiome. They show that FWD alters fungal community structure and destabilizes microbial networks. Diseased plants exhibit enrichment of pathogenic fungi like *Fusarium*, whereas healthy plants recruit beneficial bacteria such as *Rhizobium*. Network analyses reveal that FWD increases cross-kingdom interactions and reduces negative correlations, thereby undermining microbial stability. These findings advance our understanding of microbiome assembly under pathogen stress and identify potential targets for sustainable disease management.

Mora-Salguero et al. compare fertilization strategies and show that combining organic amendments, such as biowaste compost or farmyard manure, with anaerobic digestate helps sustain soil organic carbon, enhances nutrient content, and distinctly stimulates microbial communities compared to mineral fertilizer. Fungal communities responded more strongly than prokaryotic ones to fertilization regimes, underscoring how input quality and nitrogen source jointly shape microbial structure and support soil health.

Yang et al. report that long-term continuous cropping of tobacco reduces most soil nutrients (except phosphorus and manganese), alters enzyme activities, and shifts the rhizosphere microbiome toward a “fungal-type” structure. Correlation network and metagenomic analyses identify readily oxidizable organic carbon, available iron, and urease activity as key drivers reshaping the microbiome, suppressing beneficial functions such as iron respiration and carbon fixation, while enhancing plant-pathogen interactions. These findings elucidate the mechanisms of soil degradation under long-term monocropping and serve as an insight into sustainable soil management.

Li et al. systematically examine how long-term alfalfa planting regulates the assembly of ammonia-oxidizing microbial communities in the Loess Plateau. Extended planting durations increase the gene abundance of ammonia-oxidizing archaea and bacteria, shift microbial interactions from cooperation to competition, and emphasize stochastic processes in community assembly, highlighting the role of planting age in sustaining grassland ecosystem function.

Shiheng et al. demonstrate that γ-polyglutamic acid application enhances the restoration of urban abandoned land by improving soil structure, increasing organic matter and nutrient content, and enriching beneficial microorganisms such as *Actinobacteria* and *Devosia*. Metabolomic analysis further reveals upregulation of key metabolic pathways, supporting the role of γ-polyglutamic acid as an effective soil conditioner. This finding presents a promising biotechnological solution for combating soil degradation and revitalizing damaged ecosystems.

Chen et al. explore how abandoned iron ore mining reshapes endophytic bacterial communities in crop roots. Mining-induced soil acidification and trace element changes significantly increase endophyte diversity and richness, with functional predictions indicating enrichment of stress-responsive genes, offering a scientific basis for ecological restoration in mining-affected areas.

Jiamin et al. establish that soil texture, rather than water management, primarily regulates aerobic bacterial communities in Chinese croplands. Using the *gltA* gene as a marker, they show that higher clay content reduces bacterial abundance and diversity, and identify distinct ecological strategists as biomarkers, underscoring the importance of soil physical structure in microbial community assembly.

Wang Z. et al. reveal that selenium and co-occurring heavy metals are the dominant drivers of microbial structure and assembly in contaminated mining soils. Metal stress enriches tolerant taxa in Proteobacteria and Actinobacteria, reduces diversity, simplifies networks, and shifts community assembly from stochastic to deterministic processes, highlighting the profound impact of trace elements on soil ecology.

Sibalekile et al. comprehensively analyze glyphosate's interactions with soil microbial communities in genetically modified cropping systems, noting contrasting diversity outcomes shaped by soil type, application rates, and metagenomic methodologies. They call for standardized molecular and bioinformatic approaches to clarify glyphosate's impact on microbial balance.

Zhu et al. demonstrate that vegetation degradationis the dominant force structuring fungal communities rather than elevation in alpine meadows of the Qinghai-Tibetan Plateau. Degradation increases fungal diversity but reduces the complexity and stability of co-occurrence networks, linking microbial shifts directly to soil organic matter loss, which emphasizes the need for vegetation conservation.

Collectively, these studies substantially advance our mechanistic understanding of soil biological health. They reveal how specific abiotic factors, from microelements and soil texture to pollutants and management practices, govern microbial community structure, function, and stability. Beyond academic insights, this knowledge provides a scientific foundation for addressing grand challenges in global agriculture and land restoration. By deciphering the microbial basis of soil sickness, nutrient imbalance, and ecosystem degradation, this Research Topic equips us with the tools to transition from generalized farming to precision microbiome management. These contributions underscore that safeguarding soil biodiversity is not merely an ecological concern but a prerequisite for achieving food security, climate resilience, and sustainable development worldwide. The path forward lies in harnessing the invisible life beneath our feet to cultivate visible resilience above it.

